# Establishment and Characterization of the First Chinese National Standard for Nucleic Acid Amplification Technology Assays for Hepatitis E Virus Nucleic Acid Detection

**DOI:** 10.3390/pathogens12101195

**Published:** 2023-09-26

**Authors:** Manyu Li, Yan Wang, Kejian Li, Xiaotian Hao, Haiwei Zhou

**Affiliations:** 1 Division I of In Vitro Diagnostics for Infectious Diseases, Institute for In Vitro Diagnostics Control, National Institutes for Food and Drug Control, 2 Tiantanxili Rd, Dongcheng District, Beijing 100050, China; 2 Department of Hepatobiliary and Pancreatic Surgery, First Hospital/First Clinical College of Shanxi Medical University, Taiyuan 030000, China

**Keywords:** hepatitis E virus, nucleic acid detection, national standard, collaborative study

## Abstract

The detection of hepatitis E virus (HEV) RNA is the gold standard for HEV infection diagnosis. In order to address the quality control requirements for HEV RNA detection kits within China, we aimed to establish the first Chinese national standard for HEV RNA detection through a collaborative study. The candidate standard was quantified using digital PCR (dPCR). A total of five laboratories were invited to determine the estimated mean value of this national standard relative to the World Health Organization International Standard (WHO IS). Additionally, four commercial kits were used to assess the applicability of the candidate standard. The stability was determined by freeze–thaw cycles and storage at 37 °C, 25 °C and 4 °C. The estimated mean value of this national standard relative to the WHO IS was 5.67 log_10_ IU/mL. Two out of the four commercial kits can detect as low as the estimated limit of detection (LOD). The degradation rates of samples in the stability study ranged from 4% to 19%. In conclusion, we have established the first Chinese national standard for HEV nucleic acid detection against WHO IS, which can be employed to evaluate the quality of HEV RNA detection kits.

## 1. Introduction

Hepatitis E virus (HEV), which is one of the main pathogens causing acute hepatitis, leads to approximately 20 million HEV infection cases and 70,000 deaths worldwide annually [1]. HEV is classified as a single-stranded RNA virus belonging to the genus Orthohepevirus in the family Hepeviridae [1]. There are at least eight genotypes of HEV (HEV1-8), with HEV1-4 being primarily associated with human infections [2]. HEV1 and 2 are transmitted through the fecal–oral route, causing outbreaks in developing regions [1]. HEV3 and HEV4 are endemic in developed regions and transmitted by contaminated food and blood transfusion [1]. In China, the most prevalent genotypes are HEV4 and HEV3 [3]. HEV has also been recognized as a zoonotic pathogen, whose animal reservoirs include swine, rabbits, sheep and camels [4].

HEV is mainly transmitted by the fecal–oral route, usually causing self-limited disease with a low mortality rate in most immunocompetent individuals [1]. The nonspecific symptoms include loss of appetite, nausea, abdominal pain and jaundice [2]. However, in immunocompromised individuals such as organ transplant recipients, cancer patients undergoing chemotherapy and those with acquired immune deficiency syndrome (AIDS), HEV infection can progress to chronic hepatitis E, liver cirrhosis and even liver cancer [5]. Generally, the mortality rate of HEV infection is only 0.2–1% [6]. HEV infection during pregnancy can result in a mortality rate of up to 25%, with many adverse pregnancy outcomes including premature rupture of membranes, miscarriage and stillbirth [6].

The diagnosis of HEV infection includes direct and indirect diagnosis [7]. The direct diagnosis mainly includes the detection of HEV RNA and HEV capsid antigen, and the indirect diagnosis mainly includes the detection of anti-HEV IgM or IgG antibody in serum [7]. The detections of anti-HEV IgM and IgG antibodies indicate acute infection and past infection, respectively [2]. However, in immunocompromised patients, their anti-HEV antibodies can be negative due to immunosuppression [2,7]. HEV RNA can be detected in blood and feces during the incubation period, which can persist for approximately 4 to 6 weeks [2]. HEV detection in blood, feces or other bodily fluids is the gold standard for the detection of HEV infection [2]. Studies have shown that HEV can be transmitted via blood transfusion [8,9,10] and some countries such as the UK, France and the Netherlands have implemented routine or selective screening of blood donors for HEV RNA [11]. Thus, HEV RNA detection is important in diagnosing HEV infection and blood donor screening.

Currently, several commercial HEV RNA detection kits are available in China. As the gold standard for diagnosing hepatitis E, HEV RNA detection is essential and cannot be replaced in certain cases, especially in immunocompromised patients. Thus, there is an urgent need to establish a national standard to ensure quality control of these kits. In this study, we established the first Chinese national standard for HEV RNA detection calibrated against the World Health Organization International Standard (WHO IS) by collaborative study.

## 2. Materials and Methods

### 2.1. Preparation of Candidate Standard

The candidate standard was collected from the feces of an HE patient in the hospital. The fecal samples were prepared as previously described [12]. In brief, fecal samples were homogenized in sterile phosphate-buffered saline (PBS) to make 10% fecal suspensions (wt./vol.). The suspensions were then clarified by centrifugation. After being heat-inactivated at 56 °C for 30 min, the samples were stored at −80 °C pending use. The total volume of the 10% fecal suspensions was 300 mL and each aliquot had a volume of 0.5 mL. The standard was provided as fecal suspension. Our study was approved by the First Clinical Medical College, Shanxi Medical University (2021K-K157).

Total RNA in serum and fecal suspension was extracted by using QIAamp Viral RNA Mini Kit (Qiagen, Hilden, Germany), according to the protocol of the manufacturer. Real-time fluorescence quantitative PCR (RT–qPCR) in this study was performed by using the Taqman probe detection method and QuantiTect^®^ Probe RT–PCR Kit (Qiagen, Hilden, Germany) (Forward primer: 5′-GGTGGTTTCTGGGGTGAC-3′; Reverse primer: 5′-AGGGGTTGGTTGGATGAA-3′; Probe: 5′-TGATTCTCAGCCCTTCGC-3′) [13]. The reaction conditions for the one-step RT–qPCR reaction were as follows: 30 min at 50 °C, 15 min incubation at 95 °C, and 45 cycles of 10 s at 95 °C, 20 s at 55 °C and 15 s at 72 °C. The samples were considered HEV RNA positive when their Ct values were <40 in at least 2 replicates in the RT–qPCR assay.

The PCR products were then confirmed by sequencing (Sangon, Shanghai, China). Then, we used BLAST (https://blast.ncbi.nlm.nih.gov/Blast.cgi, accessed on 18 September 2023) to compare the sequence data and found that it had 99% identity with the JTK-Fukuo12C strain (GenBank ID: LC406611). Additionally, we compared the sequence data with the WHO IS (GenBank ID: AB630970) and the results showed that it had 86% identity with the WHO IS. To further confirm the genotype, reference sequences were downloaded from GenBank. Then, nucleotide sequences were aligned and analyzed by the MEGA X software package (version X, www.megasoftware.net, accessed on 1 May 2023). The alignment of all sequences was performed by Clustal X of Mega X software and then amended manually. The phylogenetic tree was constructed by the maximum likelihood method in MEGA X. One thousand bootstrap replicates were used to calculate the percentages of the branches obtained. Bootstrap values that were more than 70% were shown and were regarded as evidence of a phylogenetic grouping (Supplementary Figure S1). The results showed that its genotype belonged to HEV3, subgenotype 3b.

### 2.2. Homogeneity of Candidate Standard

Eleven vials of the candidate standard (200 vials in total) were randomly selected and tested by RT–PCR as previously described [13]. Each sample was tested in triplicate. The Ct values of eleven samples were compared by ANOVA.

### 2.3. Digital PCR (dPCR) Detection

Two independent laboratories participated in this dPCR comparative study. The labs used their own dPCR assays to extract nucleic acids twice and each experiment was performed in triplicate. Their instruments included OsciDrop^®^ Flex (Dawei bio, Beijing, China) and MicroDrop-100A^®^-100A (Forevergen, Guangzhou, China). The copy number concentration of the candidate standard was reported by every lab.

### 2.4. Collaborative Study

The National Institutes for Food and Drug Control (NIFDC) organized this collaborative study. The aim of this collaborative study was to determine the potency of this candidate standard by calibration against the WHO IS for HEV RNA NAAT assays in independent laboratories. A total of 5 labs were invited to participate in the study. All participants used their own in-house assays by their in-house reagents according to the participants’ established methods. The limit of detection (LOD) and/or limit of quantification (LOQ) should be given by each lab. The LOD is defined as the lowest amount of the analyte detectable in a single reaction. The LOQ is the lowest amount of analyte that can be quantified. Sufficient candidate standards were sent to each participant. The participants should avoid multiple freeze–thaw cycles of the candidate standard.

The participants were required to establish a standard curve by the WHO IS for HEV RNA NAAT assays and run in parallel with the candidate standard with the same dilution numbers and replicates per dilution. A total of 8 HEV RNA positive samples (4 serially diluted WHO IS and 4 serially diluted candidate standard) and 2 negative samples were tested. Using their standard HEV RNA assay, each laboratory performed three independent test runs in triplicate. Additionally, the participants were requested to report their results using standard forms provided by the NIFDC.

### 2.5. Applicability Analysis

Four commercial kits were used to assess the applicability of the candidate standard. The candidate standard was 10-fold serially diluted and tested by these kits. The serially diluted candidate standard samples around the assay endpoint were each repeated 20 times to confirm the LOD.

### 2.6. Stability and Accelerated Thermal Degradation Study

A total of thirteen vials of candidate standard were randomly selected. Each sample prior to thawing was stored at −80 °C for one week. The accelerated thermal degradation of the standard was investigated by storing 2 vials of candidate standards at 37 °C for 1d and 2d. Additionally, 3 vials of candidate standards were stored at 25 °C for 1d, 2d and 3d to assess the room temperature stability. The short-term laboratory manipulation of the standard was investigated by storing 5 vials at 4 °C for 1 d, 2 d, 3 d, 4 d and 5 d. To investigate the freeze–thaw stability, 3 vials stored at −80 °C were subjected to 1, 2 and 3 freeze–thaw cycles, respectively. The HEV RNA of them was then detected using the RT–qPCR method [13] by at least three independent assays to determine the stability.

### 2.7. Statistical Analysis

Statistical analysis was performed using the SPSS PASW Statistics v18.0 statistical software package (SPSS, Inc., Armonk, NY, USA, http://www.ibm.com/cn/, accessed on 1 May 2023). The potency of the candidate standard against the WHO IS for HEV RNA NAAT assays was calculated using parallel-line regression analysis. Data of homogeneity analysis were compared using ANOVA. A *p*-value of <0.05 was considered significant.

## 3. Results

### 3.1. Preliminary Quantification of Candidate Standard and the Homogeneity of Candidate Standard

To detect the copy number of the candidate standard, we first used the established RT–qPCR method [13], and the results showed that the copy number of it was 5.52 log_10_ copies/mL. Then, we used the standard curve established by serially diluted WHO IS to quantify the candidate standard by the RT–qPCR method [13]. The results showed that it was 5.78 log_10_ IU/mL.

The eleven vials of the candidate standard were detected for HEV RNA by RT–qPCR and each vial was tested three times. Their Ct values were compared by ANOVA. The coefficient of variation (CV) value was calculated. The results showed that there was no significant difference among the vials (*F* = 0.445, *p* = 0.645), indicating that the homogeneity of the candidate standard was good (Table 1).

### 3.2. The Copy Number of the Candidate Standard Assessed by dPCR

The copy number of the candidate standard by the RT–qPCR method [13] was 5.52 log_10_ copies/mL. To further confirm its copy number, two laboratories used the dPCR method to detect the candidate standard (Table 2). Lab 1 used primers targeting ORF3 and Lab 2 used primers targeting ORF2. The geometric mean of copy number concentration of the candidate standard was 5.59 × 10^5^ copies/mL.

### 3.3. Collaborative Study

A total of four datasets were received from four participants (Table 3). All of them used commercial kits to extract RNA. Two of them used primers targeting ORF2, one used primers targeting ORF3 and one used primers targeting a conserved region where ORF2 and ORF3 overlap.

The diluted WHO IS (3.80 log_10_ IU/mL) was used to analyze the precision and accuracy of the assays of laboratories (Supplementary Table S1). The accuracy values of the four laboratories were 103.8%, 100.0%, 103.6% and 104.5%, respectively. Their inter-assay precision was good (RSD < 5%).

All laboratories performed three independent assays and the results were presented in Table 4. Serially diluted WHO IS and candidate standards were tested and the candidate standard was quantified by a standard curve established by the WHO IS. No false positive or false negative results were reported in any of the laboratories. The R^2^ values of lab1–4 were 0.996, 0.995, 0.995 and 0.992, respectively, which were all close to 1. The results were analyzed by parallel-line regression. For each lab, the mean estimate was calculated by the results of three runs. The overall mean estimate of the candidate standard was 5.67 log_10_ IU/mL.

### 3.4. Applicability Analysis

A total of four commercial kits for HEV RNA detection were selected to determine whether their estimated LODs could be detected. The candidate standard was diluted and the serial dilutions above and below the estimated LOD were tested by four commercial kits for HEV RNA detection (Table 5). The highest concentration in the serial dilutions should be 21 times detected positive and the lowest concentration should be 21 times detected negative. The results showed that other than kit3 and kit4, kit1 and kit2 can detect as low as their estimated LODs.

### 3.5. Stability and Accelerated Thermal Degradation Results

**Figure 1:**
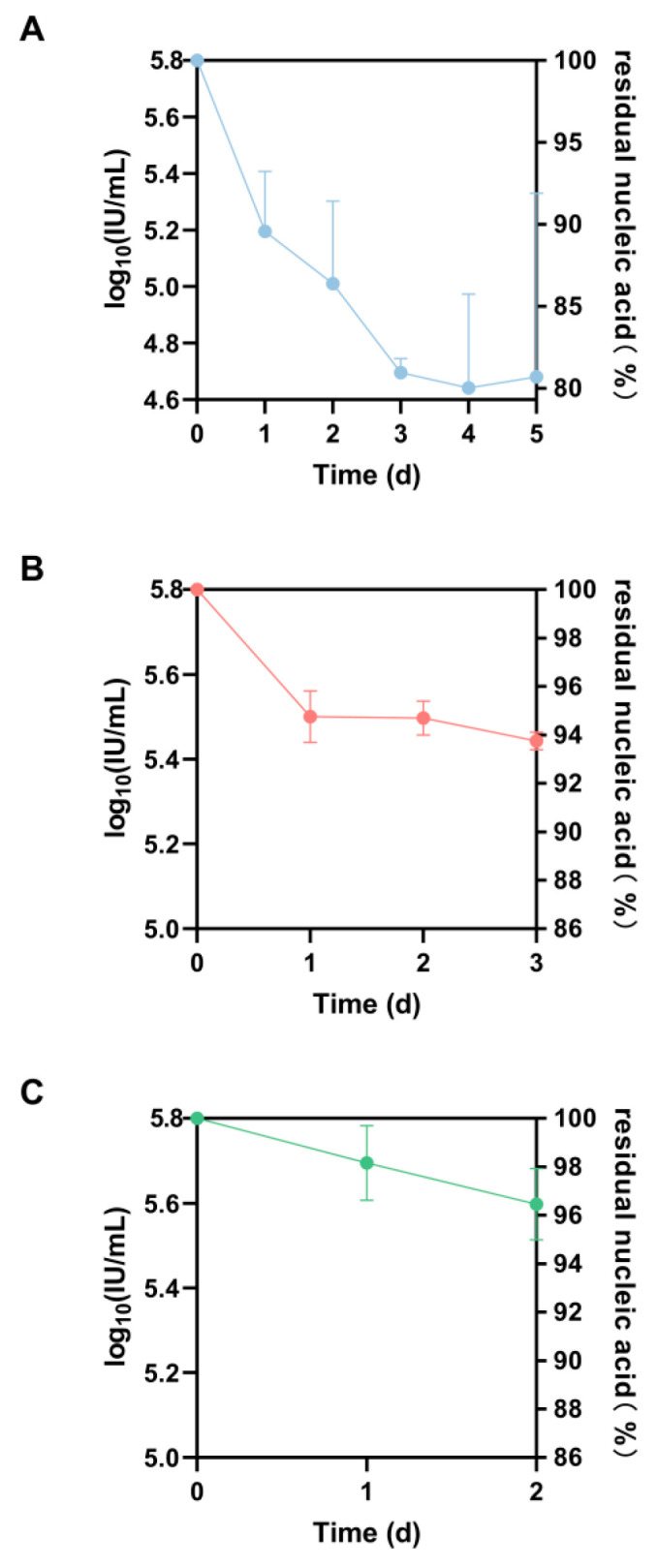
Stability degradation results after storage at different temperatures. (**A**) The degradation rates after storage at 4 °C for 1 d, 2 d, 3 d, 4 d and 5 d; (**B**) The degradation rates after storage at 25 °C for 1 d, 2 d and 3 d; (**C**) The degradation rates after storage at 37 °C for 1 d and 2 d.

Three independent assays were performed to detect the changes in the titers of the candidate standard. The residual nucleic acid content decreased to 81% after storage at 4 °C for 5 days (Figure 1A), decreased to 94% after storage at 25 °C for 3 days (Figure 1B) and decreased to 96% after 2 days in a 37 °C incubator (Figure 1C). The degradation rates of the candidate standard after 1, 2 and 3 cycles of freezing and thawing were 5%, 3% and 10%, respectively (Table 6).

## 4. Discussion

HEV is the major cause of acute hepatitis in China [2]. At present, the detection of HEV RNA is still the gold standard for the diagnosis of HEV infection, which is important for early diagnosis and blood screening [7]. Establishing a national standard for HEV RNA detection kits is critical for their quality control. The WHO has established a genotype 3a HEV strain as the IS for HEV RNA in 2013 [14]. Due to the lack of a national standard for HEV RNA detection, in this study, we aimed to establish the first Chinese national standard for HEV nucleic acid detection by collaborative study.

The selection of a candidate standard is important, and this candidate standard was chosen after several factors were considered, including total volume, genotypes and viral load. It would be beneficial to choose several candidate standards, as it allows for a better assessment of assay performance and robustness, which can be improved in future independent studies.

In this study, a collaborative study was conducted in four labs in China. The methods of RNA extraction and PCR, reagents and experiment conditions among labs were different, which may affect the consistency of their results. Previous studies have shown that automated nucleic acid extraction methods had better or similar sensitivity of PCR compared to manual extraction methods [15,16,17]. Among the four labs, lab1 used an automatic nucleic acid extraction instrument, but its sensitivity was similar to that of the others. Thus, further studies are still needed to investigate whether nucleic acid extraction methods can affect the sensitivity of HEV RNA detection. In addition, lab2 showed better accuracy, precision and lower LOD, whose primers were designed based on ORF2/3 and others’ primers were designed solely on ORF2. In previous studies, ORF1, ORF2 and ORF3 can all be used to design primers for HEV RNA detection [18,19,20]. Further investigation is required to determine whether ORF2/3-based primers can improve HEV RNA qPCR performance. In this collaborative study, the results among different labs did not vary much. The overall mean estimate of the candidate standard was 5.67 log10 IU/mL.

The genotype of the national standard belongs to HEV3, subgenotype 3b. Compared to the WHO IS, this national standard has a different matrix (plasma vs. stool) and subgenotype (3a vs. 3b), which may be considered as a factor that could affect its quantification. However, the WHO also launched the reference panel for HEV genotypes for NAT-based assays (PEI code 8578/13) for nucleic acid detection, including different matrix (serum, plasma and stool) and genotypes (1a, 1e, 3b, 3c, 3e, 3f, 3 (rabbit-like), 4c, 4g and 2a), indicating that the unitage of WHO IS can be applied to different matrix and genotypes. Whether the quantification of secondary HEV RNA standard can be affected by factors such as matrix and genotypes needs to be investigated in the future.

Then, four commercial kits for HEV RNA detection were used for applicability analysis. By testing the candidate standard, two of the four kits could detect their estimated LODs. Thus, the results demonstrated that this candidate standard is suitable to be used in the quality control and evaluation of HEV RNA detection kits under most circumstances.

The stability data showed that after different storage times, the loss in viral titers of the national standard was low. However, multiple cycles of freezing and thawing and storage at 4 °C or 37 °C should be avoided. These results suggested that the national standard was stable, but a long-term stability study is still needed in the future. In addition, more biological repeats should be included in future independent studies to further confirm the stability of the national standard.

## 5. Conclusions

In conclusion, we established the first Chinese national standard for HEV RNA detection. The estimated mean value of this national standard relative to the WHO IS (250,000 IU/mL) was 467,735 IU/mL. This standard was stable at storage temperatures of 4 °C, 25 °C and 37 °C. The implementation of this national standard will facilitate quality control and evaluation of HEV RNA detection kits, thereby promoting the development of related products in the field.

## Figures and Tables

**Table 1:** Homogeneity analysis of candidate standard.

Vial NO.	1st Experiment	2nd Experiment	3rd Experiment	*F* Value	*p* Value
1	26.63	23.30	26.01	0.445	0.645
2	22.88	21.78	22.61		
3	23.31	23.29	23.66		
4	21.11	20.92	21.11		
5	23.11	22.41	23.79		
6	22.45	22.06	22.83		
7	21.12	22.40	22.93		
8	22.41	22.30	23.10		
9	21.65	22.34	22.21		
10	25.27	25.37	25.08		
11	25.58	25.54	25.43		

Biological repeats: 11; technical repeats: 3; %CV: 6.7%, SD: 1.6.

**Table 2:** The copy number of the candidate standard assessed by dPCR (log_10_ copies/mL).

Lab NO.	1st Extraction	2nd Extraction
1	5.90 ± 0.02	5.87 ± 0.01
2	5.30 ± 0.03	5.31 ± 0.02
Geometric mean	5.59	

Biological repeats: 3; technical repeats: 3; intra-assay %CV: 0.4% (Lab 1), 0.3% (Lab 2); inter-assay %CV: 5.5%.

**Table 3:** Summary of assays used in the collaborative study.

Lab	RNA Extraction Method	Volume of Sample for RNA Extraction (μL)	Amplification Assay Method	Region of Genome Amplified	LOD/LOQ (Copies/mL)
1	Automatic Nucleic Acid Extraction Instrument (Kinghawk, Beijing, China)	200	Hepatitis E virus RNA detection kit (Kinghawk, Beijing, China) on ABI 7500 PCR System (Applied Biosystems, Foster City, CA, USA)	ORF2	1000/1000
2	QIAamp Viral RNA Mini Kit (Qiagen, Hilden, Germany)	500	Hepatitis E virus nucleic acid detection kit (Wantai, Beijing, China) on BioRAD CFX96™ Touch (Bio-Rad, Hercules, CA, USA)	ORF2/3	10/10
3	Promotor^®^ Hepatitis E Virus RNA Assay Kit (Acon, Hangzhou, China)	100	Hepatitis E virus RNA detection kit (Acon, Hangzhou, China) on ABI 7500 PCR System (Applied Biosystems, Foster City, CA, USA)	ORF3	500/500
4	Hepatitis E virus RNA detection kit (Bacme, Suzhou, China)	200	Hepatitis E virus RNA detection kit (Bacme, Suzhou, China) on Automatic Medical PCR Analysis System SLAN-96P (Hongshi, Shanghai, China)	ORF2	200/1000

**Table 4:** Results of the candidate standard by collaborative study (log_10_ IU/mL).

Dilution of Candidate Standard	Lab 1	Lab 2	Lab 3	Lab 4
1:3	5.07 ± 0.04	5.63 ± 0.07	5.71 ± 0.02	4.59 ± 0.02
1:9	4.87 ± 0.01	5.09 ± 0.12	4.97 ± 0.05	4.09 ± 0.01
1:27	4.44 ± 0.03	4.68 ± 0.07	4.23 ± 0.03	3.61 ± 0.03
1:81	4.03 ± 0.08	4.19 ± 0.17	3.48 ± 0.01	3.14 ± 0.06
Mean	5.75	6.12	5.80	5.07
Overall mean	5.67			

Serial-diluted WHO IS and candidate standard were tested and the candidate standard was quantified by a standard curve established by the WHO IS. Biological repeats: 3; technical repeats: 3; intra-assay %CV: 0.6 ± 0.4% (Lab 1), 0.3 ± 0.2% (Lab 2), 2.3 ± 0.4% (Lab 3), 0.5 ± 0.3% (Lab 4); inter-assay %CV: 9.4 ± 1.9%.

**Table 5:** Applicability analysis of four HEV RNA detection commercial kits by testing the candidate standard.

	Kit 1	Kit 2	Kit 3	Kit 4
LOD detected in this study	137 copies/mL	137 copies/mL	30 IU/mL	411 copies/mL
Estimated LOD	1000 copies/mL	500 copies/mL	10 IU/mL	200 copies/mL

Four commercial kits were selected to determine whether their estimated LODs can be detected by testing serially diluted candidate standard. Biological repeats: 3; technical repeats: 3; intra-assay %CV: 0.7 ± 0.4% (Kit 1), 1.4 ± 0.2% (Kit 2), 0.5 ± 0.2% (Kit 3), 0.9 ± 0.5% (Kit 4); inter-assay %CV: 7.9 ± 2.5%.

**Table 6:** Freeze–thaw stability analysis.

Freezing and Thawing Cycles	Log_10_ IU/mL [CV (%)]	Residual Nucleic Acid Content (%)
0	5.8 [3], *n* = 3	/
1	5.5 [6], *n* = 3	95
2	5.6 [1], *n* = 3	97
3	5.2 [5], *n* = 3	90

Biological repeats: 1; technical repeats: 3; intra-assay %CV: 2.7 ± 1.9%.

## Data Availability

Not applicable.

## Supplementary Materials

The following supporting information can be downloaded at: https://www.mdpi.com/article/10.3390/pathogens12101195/s1, Figure S1: Phylogenetic trees representing the genotype of candidate standard. Bootstrap values which were less than 70% were not shown. The ORF2 regions of the sequences were compared and shown; Table S1: Accuracy and precision of methods in different laboratories.
