# Supplementary material for: Establishment and Characterization of the First Chinese National Standard for Nucleic Acid Amplification Technology Assays for Hepatitis E Virus Nucleic Acid Detection
Source: Pathogens. 2023 Sep 26;12(10):1195. doi: 10.3390/pathogens12101195 (PMC10610164; doi:10.3390/pathogens12101195)
Supplement: Supplementary file 1 [file pathogens-12-01195-s001.zip › pathogens-2562996-supplementary.pdf]

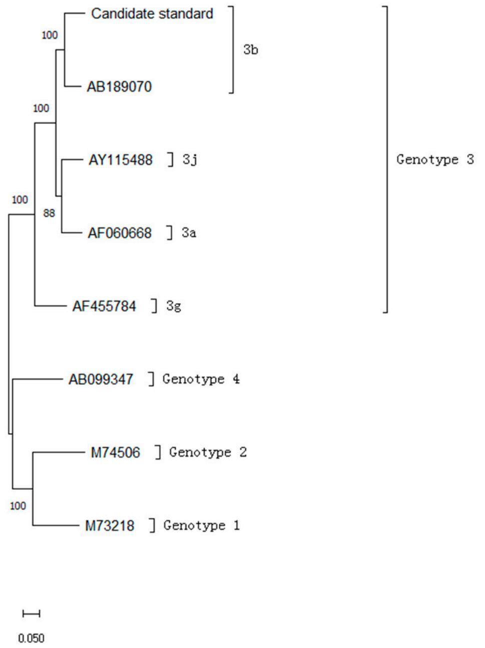

Supplementary Figure S1. Phylogenetic trees representing the genotype of candidate standard. Bootstrap values which were less than 70% were not shown. The ORF2 regions of the sequences were compared and shown.

Supplementary Table S1. Accuracy and precision of methods in different laboratories.

Test	Lab 1	Lab 2	Lab 3	Lab 4
1	3.94 log_{10} IU/mL	3.80 log_{10} IU/mL	3.90 log_{10} IU/mL	3.95 log_{10} IU/mL
2	3.91 log_{10} IU/mL	3.80 log_{10} IU/mL	3.97 log_{10} IU/mL	3.98 log_{10} IU/mL
3	3.98 log_{10} IU/mL	3.80 log_{10} IU/mL	3.94 log_{10} IU/mL	3.98 log_{10} IU/mL
Accuracy (%)	103.8	100.0	103.6	104.5
Relative standard deviation (RSD, %)	0.9	0.0	0.9	0.4
